# CDI Scores in Pediatric Psychiatric Inpatients: A Brief Retrospective Static Group Comparison

**DOI:** 10.1155/2011/134179

**Published:** 2011-06-30

**Authors:** Robert D. Friedberg, Steven A. Sinderman

**Affiliations:** ABPP, State Milton Hershey Medical Center/PSU, Penn State College of Medicine, 22 Northeast Drive, Hershey, PA 17033, USA

## Abstract

The Children's Depression Inventory is a widely researched and clinically useful measurement tool. However, research on the CDI is limited by an overreliance on outpatient samples. This is unfortunate because the CDI holds potential for use in inpatient settings. *Method*. This retrospective static group comparison examined the CDI total scores contained in 69 pediatric psychiatric inpatients treated at a large academic medical center. Patients were sorted into static groups (depressive spectrum, nondepressive spectrum) based on their diagnoses at admission. *Results*. Independent *t*-tests revealed that the CDI total scores discriminated between patients presenting with depressive spectrum disorders and youngsters admitted with non-depressive disorders. *Conclusion*. The results suggested that the CDI is a rather dimensional measure, which reflects broad negative affectivity as well as particular depressive symptoms in pediatric psychiatric inpatients.

## 1. Introduction

Childhood depression is a common psychiatric disorder and seems to be occurring earlier in children's lives [1–3]. Late childhood appears to be the typical age of onset of depressive disorders in childhood [4]. Kovacs et al. [4] found that the average age for first onset of Major Depressive Disorder was 10.98 years and the mean age for first onset Dysthymic Disorder was 8.71 years. Moreover, childhood depression was associated with alcohol and drug use in adolescence and seemed to precede the substance use by 4.5 years [2]. Unfortunately, few children receive appropriate assessment and intervention services. Seventy-five per cent of children at risk for depression are left untreated [1]. Specific assessment measures used in a sophisticated manner can facilitate appropriate symptom identification and subsequent treatment.

The Children's Depression Inventory (CDI) [5] is a widely used measure of depression demonstrating solid psychometric properties [6]. Moreover, there are several compelling reasons to use the CDI with an inpatient pediatric population. First, the CDI is a quick, easy to complete measure with strong psychometric properties. Second, scores reflect young people's subjective degree of dysphoric mood and pessimism. Pessimism influences the response to both psychosocial and pharmacological interventions. The CDI also yields important information on suicidal ideation and intent which is so important during hospital stays. Finally, the CDI is a helpful method to track progress and response to treatment.

There is some consensus in the literature that the CDI discriminates clinically depressed children from their nonclinically distressed peers [7]. Moreover, there have been several early studies examining the CDI with psychiatric inpatient children [8–11]. Kazdin et al. [10] found that depressed children had higher CDI scores than the other diagnostic groups and children with conduct disorders scored lower on the CDI than children without conduct disorders. Similarly in a subsequent study, Kazdin and his colleagues [9] found that the CDI scores for depressed children were higher than the CDI scores for children with other diagnoses. Carey et al. [8] concluded that the CDI discriminated between clinical and nonclinical populations but was unable to discriminate between clinical diagnostic groups. Nelson et al. [11] failed to find differences on the CDI between inpatient children with an affective disorder and inpatient children with a conduct disorder. 

Craighead et al. [12] concluded from their study that reliable assessment of depression in inpatient adolescents could be obtained via CDI Total Scores. In a large study of inpatient children, Liss et al. [13] found that the CDI discriminated between depressed and aggressive children. In a very recent study, Timbremont et al. [14] found the CDI total score was both predictive of a depressive disorder and able to discriminate between depression, anxiety, and disruptive behavior disorder.

In summary, several authors argue that the CDI is limited by its lack of discriminant validity [7, 15] while other authors contend the CDI enjoys adequate discriminative power [13, 14]. Such absolutistic reasoning about the measure may be unproductive because the question is a not a categorical but rather a dimensional issue. The CDI may be useful for some children to tap overall level of general distress, and for others, it provides a narrow band assessment of the particulars of their depressed mood. This study represents an effort to add clarity to this issue. 

Age, gender, and ethnicity differences represent additional critical cultural vicissitudes in clinical research and practice. Therefore, age, gender, and ethnicity differences are important variables to consider when studying children's depression. Gender and age differences in depression have been studied extensively with mixed results and conclusions. Kovacs [6] concluded that studies examining gender and age differences on the CDI were inconsistent showing no clear age or gender effects. In a very recent study, girls scored higher than boys on the CDI [16].

Some researchers have discovered age × gender interactions reflecting the finding that the prevalence of depression increases for girls during adolescence but not for boys in this age period, whereas other investigators have failed to find these age × gender trends [17]. Nolen-Hoeksema and Girgus [3] also noted that there are age and gender changes in children's explanatory styles. Prepubertal girls are more optimistic than prepubertal boys. Pessimism rises for both boys and girls through mid-adolescence, but boys become more optimistic than their female counterparts by late adolescence.

 Moreover, Twenge and Nolen-Hoeksema [17] reported that studies exploring ethnic differences also show mixed findings with some investigators discovering differences between ethnic groups on the CDI and others reporting no differences. Roberts [18] concluded that Hispanic children are at risk for depression. The U.S. Surgeon General's Report [19] on mental health stated there is a high risk of suicide in Hispanic youth. In their own meta-analysis, Twenge and Nolen-Hoeksema [17] similarly found that Hispanic children endorsed more depressive symptoms than either African-American or Caucasian children. Gibbs [20] was also alarmed by the increase in suicide rates for African American youth. Accordingly, due to the mixed findings of the previous research, the broad recommendation of the American Psychological and American Psychiatric Association to regularly include these variables in research, and common sense clinical and methodological reasoning, the present study also explored age, gender, and ethnic differences on the CDI.

 This project extends the literature by examining CDI scores in inpatients since most other studies investigating CDI scores are completed with outpatients. In an attempt to clarify the issue of the CDI's discriminative value, the present work aims to discern whether CDI scores differ in children diagnosed with depressive spectrum or non-depressive spectrum disorders. Additionally, the project seeks to add clarity to the ambiguous findings regarding the relationship between CDI scores and ethnicity, age, and gender. Accordingly, the present study posed several hypotheses. First, it was predicted that children's CDI total scores will be higher for patients initially hospitalized for primarily mood spectrum disorders than for patients hospitalized for nonmood spectrum disorders as suggested by Liss et al. [13] and Timbremont et al. [14]. Additionally, due to the inconsistent findings from the previous literature [6, 16–20], exploratory hypotheses regarding age, gender, and ethnicity and their relationship to CDI scores were developed. More specifically, it was predicted that older children will endorse more symptoms on the CDI than younger children. Girls were expected to score higher on the CDI than boys. Euro-American children were expected to score higher on the CDI than their nondominant culture peers. Finally, a further exploratory hypothesis predicted that greater CDI total scores would be significantly associated with longer lengths of stay in the hospital.

## 2. Method

### 2.1. Procedure

The study is a posthoc static group comparison and was approved by Institutional Review Board (IRB) as an exempted archival study. Due to the archival retrospective nature of the static group comparison design and that the fact data was completely anonymous, the IRB approved the research as exempt from requiring written consent. 

 Inpatient children's data (age, gender, ethnicity, CDI scores, diagnoses, length of stay) were obtained via archival methods from the charts of a child psychiatric inpatient unit in a large academic medical center located in a rural section of the eastern United States. The psychiatric unit housed 16 beds and generally served patients who were depressed, disruptive, and/or a danger to themselves, others, or otherwise grossly impaired. Typical criteria for admission emphasized danger to self or others. Data were collected from a two-year period between April 2002 and April 2004. Only children whose charts contained a CDI and admitting diagnoses were included in the static group comparison. 

As is typical in a retrospective study, there was not a standard protocol given to every inpatient. Thus, some children admitted to the unit did not receive the CDI. Due to the limitations of the retrospective data retrieval process, it was impossible to precisely determine how many children did not receive the CDI while they were hospitalized or the reason they were not given the measure. There was no missing data for the 69 patients.

### 2.2. Classification of Groups

 Static group classification was based on the admitting diagnosis found during the chart review. These diagnoses were originally determined by child psychiatric fellows and their supervising attending psychiatrists (*n* = 3) during the patients' stay on the unit. One psychiatrist accounted for 45% (*n* = 31) of the diagnostic entries, a second psychiatrist accounted for 37% (*n* = 23), and a third psychiatrist contributed 22% (*n* = 15) of the diagnoses

As previously mentioned, the admitting diagnoses were obtained from the retrospective chart review and subsequently sorted into two groups: depressive spectrum (*n* = 34) and nondepressive spectrum (*n* = 35) diagnoses. Patients who carried any type of depressive spectrum diagnosis were placed in the depressive spectrum disorder group. This group then included depressive disorders and any accompanying conditions. The non-depressed group included all diagnoses **except **depressive spectrum disorders. Table 1 shows the type and number of diagnoses represented in each group. It should be noted that the individual totals exceed the total numbers in each group due to the inclusion of comorbid conditions.

### 2.3. Instrument

The CDI is a 27-item self-report inventory of childhood depression that taps a variety of depressive symptoms. Items assess negative mood, interpersonal difficulties, negative self-esteem, ineffectiveness, and anhedonia in children ages 7–17 years of age. Each item offers respondents three alternatives scored 0, 1, or 2 and accordingly raw scores range from 0 to 54. Several studies [6, 15, 21] recommended 13 as a cutoff score for clinical populations and 19 as the threshold for community samples in the United States. More recently, 16 has been recommended as a cut-off for Dutch samples [14, 22]. Kovacs [6] reports alpha reliability coefficients ranging from  .71 to  .86.

## 3. Results

### 3.1. Descriptive Characteristics of the Inpatient Sample

69 patients' charts that contained a completed CDI and diagnoses were reviewed (Male *n* = 52; Female *n* = 17). The mean age of the patients' was 10.9 years with an age range of 7 to 15 years of age. The sample was predominantly Euro-American (*n* = 47) with a small number of African American (*n* = 15) and Hispanic American (*n* = 7) patients. Due to the small numbers, ethnic minorities were grouped into a nondominant culture category (*n* = 22) for data analysis. The patients' length of stay was also culled from the chart. The average length of stay was 7.59 days with a standard deviation of 4.89 days.

### 3.2. Differences on the CDI

An independent sample *t*-test compared the CDI total scores for the mood disorder and nonmood disorder inpatient children. As predicted, the results indicated a significant difference between the two groups of children on the CDI (*t* = 3.75, df = 67, *P* < .001). More specifically, the depressive spectrum children scored higher on the CDI (Mean CDI = 21.18; SD = 9.35) than their non-depressive disorder counterparts (Mean CDI = 13.66; SD = 7.21). Cohen's **d** suggested a large effect size (*d* = .91).

As predicted, CDI total scores were significantly correlated with children's age (*r* = .24, df = 67, *P* < .05). The results indicated that older children endorsed more symptoms on the CDI. Independent *t*-tests revealed no significant differences for gender in the total scores (*t* = .60, df = 67, *P* = ns). Additionally, no differences were found for ethnicity in the total scores (*t* = −.51, df = 67, *P* = ns). To test the relationship between admission CDI total scores and Length of Stay (LOS), Pearson product moment correlations were computed. The results revealed no statistically significant relationships of LOS with the CDI total (*r* = .04, df = 67, *P* = ns).

## 4. Discussion and Implications

As hypothesized, child psychiatric inpatients with mood spectrum disorders were higher on the CDI total score than their peers diagnosed with non-mood spectrum disorders. Thus, it appears the CDI adequately captures inpatient children's depressive symptoms and may be able to discriminate between mood disordered children and their non-mood disordered counterparts. However, a logistic regression would be necessary to fully test and substantiate this finding. The children's age was significantly correlated with the CDI total score indicating that older children endorsed more symptoms than younger children. Thus, these findings emphasize the importance of using appropriate age norms and cut-offs with CDI.

Mood disordered inpatients' mean scores on the CDI exceeded the cut-offs recommended for both clinic-referred (Raw Score = 13) and community (Raw Score = 19) populations in the United States [2, 7]. The non-mood disordered group Mean CDI (13.66) also exceeded the cutoff score for clinic samples. Additionally, the mean scores for the two groups were remarkably similar to the results obtained in the Liss et al. study [13]. In the Liss et al. study, the mean for the depressed group was 19.17 compared to the 21.18 in the present study. The non-mood disordered group mean in the Liss et al. study was 14.55 compared with the 13.66 obtained in the non-mood disordered group in the present study.

The relatively high scores and similarity between the inpatients in the two studies yields some interesting implications. First, the similarity between the scores obtained in two different inpatient samples in different geographic regions adds to the confidence regarding the generalizability to other inpatient settings. Second, the CDI appears to measure significant negative affectivity associated with the children's inpatient hospitalization. When the threshold for hospitalization is met, children are clearly emotionally distressed regardless of their reason for admission. The mood-disordered children may have higher levels of dysphoric mood but the non-mood-disordered children are also clearly experiencing considerable negative affects (e.g., sad, anxious, angry moods). 

This finding seems to make sense in the context of the previous empirical and clinical literature. The CDI has been shown to assess both negative affectivity as well as conduct and behavior problems [5]. The CDI scores revealed relatively high levels of dysphoria in both the mood and non-mood disorders groups. Hospital stays themselves may represent stressors for young patients thereby increasing their depressed mood [23]. Inpatient hospitalizations involve a separation from patients' living environments. This separation may result in a loss of reinforcement from various sources (e.g., children, parents, friends, partners, etc.). Moreover, they are placed in an unfamiliar environment. They may also perceive the hospitalization as a form of coercive control, abandonment, and signs they are hopeless. All these attributions can fuel increased depressed moods. 

There was not a significant correlation between CDI scores at admission and length of stay on the unit. It is probable that factors others than inpatients' CDI scores predicted length of stay. As previously mentioned, the average length of stay was 7.59 days with a standard deviation of 4.89 days. Thus, the length of stay was relatively short with limited variability. In the current climate of short inpatient stays, market and economic forces may be more determinative of length of stay than initial level of self-reported depression. 

The lack of gender differences is interesting. The CDI manual itself reported mixed results regarding gender differences [6]. In general, previous research examining gender differences in childhood depression shows that adolescent girls have higher rates of depression than teenage boys [3]. However, prepubertal boys and girls report similar levels of depressive symptoms [3]. Thus, the age of the inpatients may have been a factor in attenuating the gender differences. The present sample was relatively young. Additionally, there were considerably less female (*n* = 17) inpatients in the study than male (*n* = 52) inpatients. Accordingly, the sample size may have truncated the differences. Finally, it is possible that once the clinical threshold for admission is crossed, gender differences are minimized [24].

This study was a static group comparison and suffers from several limitations. While static group comparisons control well for testing, instrumentation, and regression to the mean, they are also vulnerable to validity threats from selection and attrition. [25]. Similar to the Kovacs et al. [4] study, diagnoses were determined by psychiatric semistructured interviews with patients and their families. While structured diagnostic interviews would be ideal to determine diagnosis, they were not part of the routine care delivered at the time of the retrospective study. However, the diagnostic process seems to approximate the way diagnoses are determined in most nonresearch clinical settings. Forty-five per cent of the diagnoses were determined by one psychiatrist. Reliability data on the diagnoses was not able to be collected so a potential diagnostic bias must be considered and suggests a more important limitation of the study. Accordingly, adding checks on diagnostic reliability would be a meritorious future research endeavor. 

The sample size and use of a single setting are additional limitations of this study. The sample was relatively small and included few females and children of color. Thus, the failure to find significant differences may reflect a truncated sample size rather than a lack of actual differences. All the patients came from a single academic medical center. Although the medical center serves a large geographic region, which encompasses rural and urban centers, statements about generalizability should be conservative. Further, this study examined the CDI in inpatients and did not include an outpatient control group. Therefore, no conclusions about the CDI in outpatients are appropriate. 

The chart review did not contain data from multiple informant sources. Additionally, CDI data was not available at discharge so the only available CDI data was obtained at admission. Comparing changes in the CDI at admission and discharge for the two groups would be interesting. Additionally, analyzing differences in admission and discharge scores with length of stay data would also be a compelling research direction. Developing a multiple regression equation including several predictor variables to examine factors, which reliably determine length of stay is an important future project. Continued research studying the CDI with inpatients and outpatients will expand our theoretical and clinical knowledge base.

## Figures and Tables

**Table 1:** Type and frequencies of diagnoses represented in each static group.

Depressed group	*n*	Nondepressed group	*n*
Depressive disorder NOS	19	ODD	20
Major depression	7	ADHD	16
Adjust disorder with Dep Mood	2	Pervasive Dev disorder	4
Mood disorder NOS	3	PTSD	4
ADHD	8	Psychosis NOS	2
ODD	5	Reactive attachment	2
OCD	2	Separation anxiety	1
Substance abuse	2	Anxiety NOS	1
PTSD	2	OCD	1
Bipolar disorder	1	Conduct disorder	1
Psychosis NOS	1	Eating disorder NOS	1
